# Anti-diabetic Potential of a Stigmasterol From the Seaweed *Gelidium spinosum* and Its Application in the Formulation of Nanoemulsion Conjugate for the Development of Functional Biscuits

**DOI:** 10.3389/fnut.2021.694362

**Published:** 2021-09-16

**Authors:** Navya Poulose, Arya Sajayan, Amrudha Ravindran, Ambili Chandran, G. Balasubramanian Priyadharshini, Joseph Selvin, G. Seghal Kiran

**Affiliations:** ^1^Department of Food Science and Technology, Pondicherry University, Pondicherry, India; ^2^Department of Microbiology, Pondicherry University, Pondicherry, India

**Keywords:** stigmasterol, antioxidants, antidiabetic, nanosized, *Gelidium spinosum*, glycogen synthesis

## Abstract

The seaweed *Gelidium spinosum* was selected for the extraction of phytosterol by the Soxhlet method. The extracted phytosterol was chemically characterized as stigmasterol using Fourier-transform infrared spectrometry and gas chromatography–mass spectrometry analysis. The antioxidant and α-amylase inhibitory activity of stigmasterol has been confirmed by *in vitro* assays. The *in vivo* studies demonstrated an anti-diabetic effect in streptozotocin (STZ)—induced hyperglycemic rats. Biochemical analysis showed administration of stigmasterol reduced the blood sugar, urea, and creatinine level. The stigmasterol was then nano-emulsified and incorporated into dough for biscuit formulation. The stigmasterol incorporated biscuit showed higher proximate values, low moisture content, lighter color and the textural property revealed lower hardness. Sensorial results showed acceptability when compared to the control. This study demonstrated the stigmasterol reduced hyperglycemic effects and therefore could be used as a supplement in diets for diabetic patients.

## HIGHLIGHTS

- Stigmasterol isolated from *Gelidium spinosum* showed potent α amylase- inhibitory activity.
- The *in vivo* animal study showed stigmasterol reduce the blood sugar level in hyperglycemic rats.
- Nano-emulsified stigmasterol was incorporated into biscuit and its textural, sensorial and proximate analysis of the biscuits was determined.

## Introduction

Seaweeds (macroalgae) constitute a significant source of bioactive compounds and can be exploited to yield a great variety of metabolites for pharmaceutical, cosmetics, food, and feed supplements. Bioactive compounds from seaweeds have a wider implication in pharmaceutical and medical scenarios. They are a rich source of antimicrobial, antibiotics, anticancer, antiviral compounds, antidiabetic, anti-inflammatory, immunomodulatory effects, and also protect the cardiovascular system (1). The health benefits of seaweed gained attraction in the nutraceutical industry. The bioactive peptides from seaweed are antihypertensive agents by inhibiting angiotensin-converting enzyme (ACE) (2). Seaweeds are grouped based on their unique photosynthetic pigments, which give them their characteristic colors and unique properties. They are also rich in polysaccharides such as alginates, carrageenan fucoidan and laminarans and are likewise considered dietary fibers (3). Consumption of algal fiber helps in the development and protection of intestinal microflora in humans.

Phytosterols are a class of sterols principally synthesized by plants and algae and cannot be synthesized in the human body. Phytosterols are essential constituents of the cellular membrane, maintain membrane fluidity, stabilize phospholipid bilayers, and play an important role in signal transduction (4, 5). The type of seaweed, geographic origins and developmental phases of the living beings can add to various phytosterol profiles (6). Studies reported fucosterol is the most abundant sterol present in brown algae (7–10). The great diversity of marine algae may consequently be an exceptionally source of structurally different phytosterols. Phytosterol reduces LDL cholesterol and is essential to maintain the good cholesterol. It protects the heart from cardiovascular diseases. The European Food Safety Authority recommended that phytosterol consumption of 1.5–2.4 g/day reduces the risk of heart disease (11).

This study intends to explore phytosterols from the seaweed *G. spinosum* and the purified sterol was identified as stigmasterol. The extracted stigmasterol showed potent anti-diabetic activity on rats, the blood serum and biochemical analysis evident the anti-diabetic effect. Hence the stigmasterol was nano-emulsified and incorporated into the biscuit dough for the preparation of biscuits. The prepared stigmasterol incorporated biscuit showed improved textural quality including reduced hardness and fracturability, color and sensorial attributes compared to the control. Thus, the stigmasterol can be supplemented with food and can be used as an alternative strategy to treat diabetes.

## Materials and Methods

### Seaweed Collection and Processing

The Seaweed *Gelidium spinosum* was collected from Mandapam coast, Tamilnadu, India. Collected seaweed was washed thoroughly to remove the salt, surface impurities, sand particles, and epiphytes from the sample's surface. The water was drained off and the seaweed sample was chopped and sun dried. The dried seaweed was powdered in the grinder and the samples were stored at 4°C for further studies.

### Extraction and Purification of Phytosterols

The seaweed powder was taken in a thimble holder and then placed in a distillation flask. Extraction was performed using ethanol in a Soxhlet apparatus under steam for 6 h. The extract was cooled, and then water: petroleum ether (1:1) was added to a separating funnel. The layers were collected separately and the lipid containing fractions were mixed with 1 M ethanolic KOH and stirred overnight. The mixture was diluted with distilled water and extracted with 3 parts of diethyl ether. The extract was further dried over anhydrous sodium sulfate and deactivated alumina. The presence of phytosterols was confirmed by the Salkowski test (12). The organic phase of the extracted compound was then concentrated to dryness using a rotary evaporator (Yamato DC400, Japan), quantified and then purified using silica gel column chromatography using the solvent system methanol: water (4:1) followed by reverse phase HPLC on a C-18 column with a mobile phase of 1% ethanol in hexane, eluted fractions were then used for further analysis (13).

### Characterization of Phytosterols

Thin layer chromatography was performed using the solvent system of methanol: water (4:1). The functional group of the phytosterol was determined by Fourier-transform infrared Spectroscopy (FTIR) analysis. To determine the functional groups, the analysis was carried out in the 4,000–450 cm^−1^ spectral region at a resolution of 1 cm^−1^ and 50 scans on a Perkin Elmer system. The samples were dispersed in KBr pellets the homogeneous mixture was prepared using a hydraulic press. Purified phytosterol extract was subjected to trimethylsilylation and then analyzed using gas chromatography–mass spectrometry (GC-MS). Briefly, dried extract was added with 100 μl of N-methyl-N-(trimethylsilyl)-trifluoroacetamide (MSTFA) in a React-tube. The tube was heated at 90°C for 20 min and then cooled. The mixture was further dried at 60°C and then analyzed by GC-MS (Agilent 7890A - 240 MS with Ion Trap). The peaks obtained in the GC was subjected to mass spectral analysis and the individual components were identified by comparison with standard libraries.

### Determining of Antioxidant Activity

For the antioxidant determination, stigmasterol was reconstituted in ethanol at the concentration of (100 mg/ml) and 25–500 μg/ml of extract were used for free radical scavenging activity (1, 1-diphenyl-2-picrylhydrazyl (DPPH) radical assay. Scavenging activity was calculated by using ascorbic acid as standard.


(1) $$\begin{array}{l} \%\;Inhibition\;Antioxidant\;capacity\\ =\;\frac{control\;absorbance-sample\;absorbance}{control\;absorbance}\;X\;100 \end{array}$$

The hydroxyl radical scavenging activity of stigmasterol was determined according to the protocol of (14). All the experiments were performed in triplicates. The hydroxyl radical scavenging activity was calculated by the following equation.


(2) $$\begin{array}{l} Hydroxyl\;radical\;scavenging\;activity=1-\frac{A_{1}-A_{2}}{A_{0}}\times 100\; \end{array}$$

### Alpha-Amylase and Alpha Glucosidase Inhibitory Assay

α- amylase inhibitory activity was evaluated as per the method of Jayasri et al. (15). Briefly, varying concentrations of stigmasterol (50–200 μg/ml) was added to 0.02 M sodium phosphate buffer of pH 6.9. To this 0.5 mg/ml of α- amylase was added and incubated at 25°C for 10 min. To this 250 μl of starch solution (1%) was added. After incubation of 10 min, the reaction was stopped by adding 500 μl of dinitrosalicylic acid. The absorbance of the assay mixture was recorded at 540 nm. Alpha amylase inhibitory activity was calculated as percentage inhibition using the formula,


(3) $$\begin{array}{l} \%\;Inhibition=\;\frac{Abs_{control}-Abs_{Sample}}{Abs_{control}}\times 100\; \end{array}$$

Alpha glucosidase inhibitory potential was determined by the method of Javadi et al. (16). Briefly, different concentrations of stigmasterol were used as test, acarbose was used as positive control and solvent was set as a negative control. Samples and controls were added to reaction mixture containing 100 μL of 30 mM phosphate buffer and 15 μL of α-glucosidase enzyme (0.02 U/μL) from *Saccharomyces cerevisiae* (Sigma). The control and sample mixtures were then treated with 75 μL of PNPG (ρ-Nitrophenyl-ρ-d-glucopyranosidase, 0.3 mg/mL). The amount of ρ-nitrophenol released was measured using microplate reader at 405 nm. Alpha glucosidase inhibitory activity (%) was calculated using the formula,


(4) $$\begin{array}{l} \%\;Inhibition=\left(\frac{Abs_{control}-Abs_{Sample}}{Abs_{Control}}\right)\times 100\; \end{array}$$

### Demonstration of Anti-diabetic Activity of Stigmasterol in Rats

Healthy male Sprague Dawley rats weighing between 130 and 160 g (5–6 weeks old) were procured from Biogen laboratory animal facility, Bangalore, India. The study was carried out in an air-conditioned room at an ambient temperature of about 25°C, relative humidity of 40–60% with 12 h light/ dark cycle, and provided free access to feed and water. To determine the anti-diabetic activity, the animals were grouped into four groups as given below:

Group 1: Normal control rats fed with normal pellet and sterile water orally for 4 weeks (*n* = 10).

Group 2: Rats treated with single dose of drug streptozotocin (35 mg/kg body weight) and fed with normal pellet and sterile water orally for 4 weeks (*n* = 10).

Group 3: Rats treated with single dose of drug streptozotocin (35 mg/kg body weight) and fed with normal pellet mixed with metformin 250 mg/kg body weight and sterile water orally for 4 weeks (*n* = 10). Metformin is a commonly used anti-diabetic drug and is used as positive control of (17).

Group 4: Rats treated with single dose of drug streptozotocin (35 mg/kg body weight) and fed with normal pellet mixed with stigmasterol 200 mg/kg body weight and sterile water orally for 4 weeks (*n* = 10). Three days after streptozotocin injection, the rats were examined for fasting blood glucose to confirm the diabetic stage. Fasting blood glucose was measured at 0, 7, and 14 days with glucometer (Dr. Morepen Gluco One Blood glucose monitoring system). The rats with fasting blood glucose above 200 mg/ dL were considered as diabetic. The dose of stigmasterol was selected based on the Human equivalent dose (HED) conversion to animal equivalent dose (AED) (18).

### Determination of Biochemical Parameters

After the experimental regimen, the animals were sacrificed by cervical dislocation after giving mild anesthesia using chloroform. The blood samples were drawn from retro orbital site (under anesthetic condition) without anticoagulant and serum was separated by centrifugation at 2,500 rpm. The biochemical parameters include serum protein, urea, creatinine and glycogen content was determined using Biosystems kit, India.

### Histopathological Analysis

The organs kidney and liver were dissected into small sections of 1 cm^3^ and then fixed in 10% neutral formalin and then embedded in paraffin wax. The sections were cut into thin sections of 5 μm and then stained with haematoxylin -eosin, mounted in deparaffinated xylene, dried and then visualized under microscope (17).

### Formulation of Stigmasterol Nano-Emulsion

To prepare stigmasterol nano-emulsion, 8 g of stigmasterol was added to 80 ml of water and heated at 30°C. To the mixture 6 g of lecithin and 6 ml of olive oil was added and vortexed for 5 min. The mixture was stirred using a magnetic hot plate set at a temperature of 50°C for 10 min and blended using a homogenizer at 6,000 rpm for 2 min and then fine nano emulsions were obtained using a high-pressure homogenizer. The formed emulsion was then freeze dried using a lyophilizer (Yamato, Japan). Lyophilized powder obtained was characterized for particle size analysis and its formulation into biscuit as a functional food ingredient.

### Particle Size and XRD Analysis

Particle size of the stigmasterol emulsion was determined using a Particle size analyzer and performed using a laser diffraction particle size analyzer (Beckman-Coulter, LS-230, Miami, FL, USA). The degree of crystallinity was determined using a Rigaku (30 kv/25 mA) Geigerflex D/Mac, C series diffractometer (Tokyo, Japan) with Cu-kα radiation (λ = 1.5406 A°) at room temperature in glancing inclined angle mode.

### Preparation of Biscuit

Biscuits were prepared as described in (19) with necessary modifications. The ingredients used were 100 g of whole wheat flour, 10 g of palm sugar, 50 g butter, 0.5 g baking powder and 2.0 g of egg white. In the test baking powder and egg were replaced with lyophilized powder of stigmasterol of 2.5 g. All the ingredients were mixed well and made into a smooth dough, which was then sheeted to a thickness of 4 mm and was cut using a cookie cutter. The biscuits were further baked in a preheated oven at 160°C for 20 ± 5 min. The baked biscuits were then stored in an air—tight container for further analysis.

### Proximate Analysis of Biscuit

Proximate analysis of the control and the stigmasterol incorporated biscuit was analyzed and the ash, moisture, fiber, fat, and protein content were determined by AOAC method (20). Carbohydrate content was obtained by subtraction method.

### Texture Analysis of the Biscuit

Texture of the biscuit was analyzed using a Texture analyser (TA–HDplus, Stable Micro Systems, Surrey). The biscuit samples were pressurized with the probe until the biscuit broke. A three-point bending test was conducted using a three- point bending rig (HDP/BS) at a test speed of 2 mm/s and a distance of 5 mm. Calibration was carried using a load cell of 50 kg, where the hardness of the sample was analyzed (19, 20).

### Color Analysis of Biscuit

Color analysis of the biscuits was performed using Hunter lab spectrophotometer (D-25, Hunter Associates Laboratory, Ruston, USA (21). Biscuits from each set were positioned in a glass sample cup of 5.8 cm internal diameter and were analyzed for color coordinates using L^*^, a^*^, b^*^ color space. The colors were analyzed in triplicates, and the mean was recorded.

### Sensory Evaluation of Biscuit

A total of 15 volunteers, age 23–30 (seven males and eight female) evaluated the sensory characteristics of the biscuits The samples were analyzed for appearance, color, flavor, texture, crispiness, hardness and overall acceptability using a 9- point hedonic scale for sensory evaluation, varying from 9 (like extremely) to 1 (dislike extremely) (22).

### Statistical Analysis

Statistical analysis used in this study represents average of triplicate experiments. The data were represented in terms of mean ± standard deviation. The data were analyzed using one-way ANOVA with Bonferroni posttest at *P* < 0.05 being significant using SPSS software.

## Results

### Collection and Processing of Seaweeds

In this study, the seaweed *G. spinosum* was collected from the Gulf of Mannar, Mandapam coast, and processed for phytosterol extraction. The Salkowski test showed brown ring, which confirmed the presence of phytosterols in the seaweed extract. The yield of the crude extract of phytosterol was 3.56 mg/gm.

### Determination of Antioxidant Activity by DPPH Assay and Hydroxyl Radical Scavenging Activity

**Figure 1:**
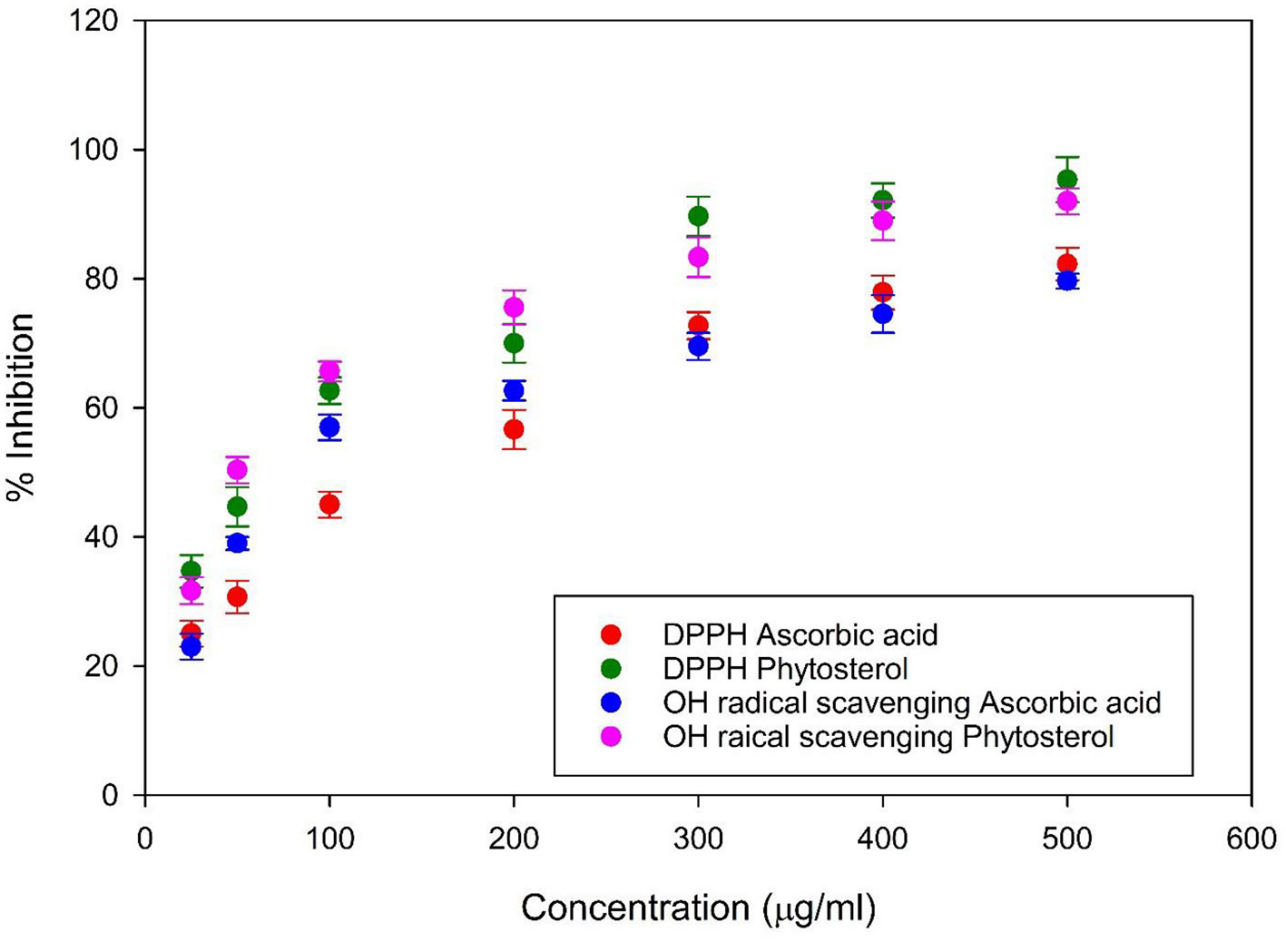
Antioxidant activity by 1, 1-diphenyl-2-picrylhydrazyl and hydroxyl radical scavenging assay. The assay results evidence that antioxidant activity increases with the concentration of the stigmasterol, and the activity was higher than the control ascorbic acid.

DPPH radical scavenging assay is a method used to evaluate the free radical scavenging activity of the seaweed extracts. The assay results showed the phytosterol of *G. spinosum* showed significant effect in inhibition of DPPH free radical up to 95.33 ± 3.51 at 500 μg/ ml (Figure 1). The obtained results indicated the scavenging activity of phytosterol was concentration dependent. Ascorbic acid showed effective concentration (EC50) of 178.07 μg/ml, while phytosterol had an EC50 of 65.28 μg/ml. The hydroxyl scavenging assay showed phytosterol exhibited the inhibition of 92 ± 2.0 and the inhibition rate was higher than the standard ascorbic acid of 79.66 ± 1.15 (Figure 1). The EC50 of hydroxyl radical scavenging activity of ascorbic acid was found to be 146.53 μg/ml, whereas, stigmasterol showed 46.53 μg/ml.

### Characterization of Phytosterols

**Figure 2:**
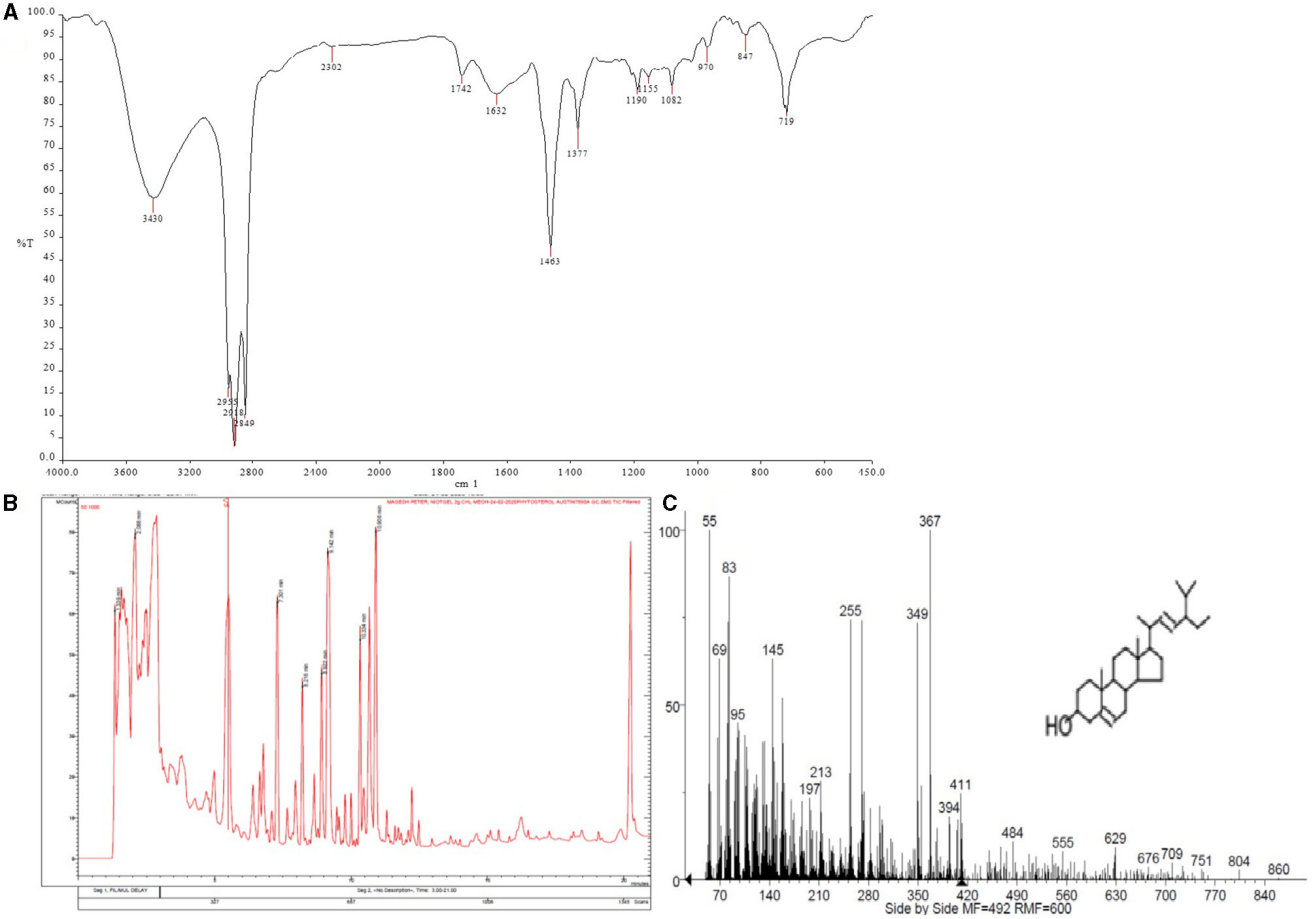
**(A)** Fourier-transform infrared spectrometry spectrum showing the characteristic peaks of stigmasterol **(B,C)** Gas chromatography–mass spectrometry spectrum showing the presence of stigmasterol.

The thin layer chromatography of the phytosterol extract showed spot in the solvent system methanol: water (4:1) with an Rf value of 0.3. FT-IR spectra showed characteristic absorption band at 3,430 cm^−1^ correspond to the -OH group. Peaks in the range of 2,955 to 2,326 cm^−1^ correspond to the presence of CH_2_ and CH_3_ groups. Band observed at 1,742 cm^−1^ may be due to the presence of carbonyl group C=O, peaks at 1,632 to 1,577 cm^−1^ corresponds to C=C and C-H vibrations, O-H bending and C-O stretching at 1,377 cm^−1^, peak at 1,055 cm^−1^ corresponds to secondary C-O vibrations. GC-MS analysis of the TMS derivatives of the extract showed the presence of stigmasterol with a retention time of 20.82 min and a mass of 412.69 g/mol (Figure 2).

### Determination of α- Amylase and α- Glucosidase Activity

**Figure 3:**
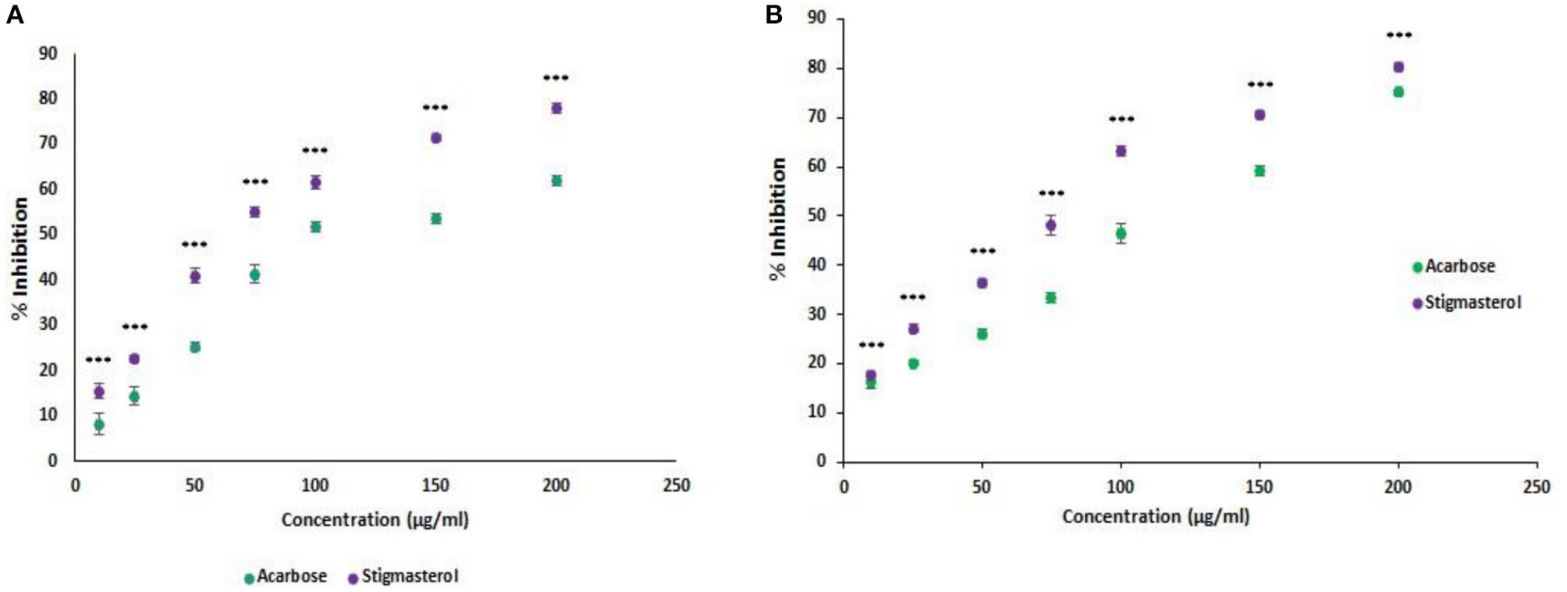
**(A)** Alpha-amylase inhibition assay of the stigmasterol. The result shows that the alpha-amylase inhibition activity increases with the concentration of the stigmasterol, and the activity was higher than the control acarbose. **(B)** Alpha-glucosidase inhibition assay of the stigmasterol. Error bars indicate standard deviations, significance ***P < 0.001.

Control of blood glucose levels is important in the control of micro and macro vascular complications. To control hyperglycemia, inhibition of starch hydrolysis was essential and stigmasterol extracted from the seaweed was found to possess α- amylase inhibitory activity. The assay results showed stigmasterol inhibited α- amylase (78 ± 1.0%), and the activity was higher when compared to the standard acarbose (62 ± 1.0%) at a concentration of 200 μg/ml (Figure 3A). Acarbose with an IC50 of 133.82 μg/ml, while stigmasterol with inhibitory concentration of 89.27 μg/ml. Alpha glucosidase mediates the breakdown of the oligo and disaccharide units of starch into glucose during digestion before it is absorbed into the bloodstream. Thus, inhibition of alpha glucosidase can be used as an ideal method to prevent diabetes. At a dose of 200 μg/ml, the stigmasterol alpha glucosidase inhibitory activity was found to be 80.3 ± 0.6%, which was higher than that of standard acarbose (75.18 ± 0.5%). The IC50 value of alpha glucosidase inhibition was found to be 89.98 μg/ml for stigmasterol and 120.18 μg/ml for standard acarbose (Figure 3B).

### Anti-diabetic Effect of Stigmasterol

**Figure 4:**
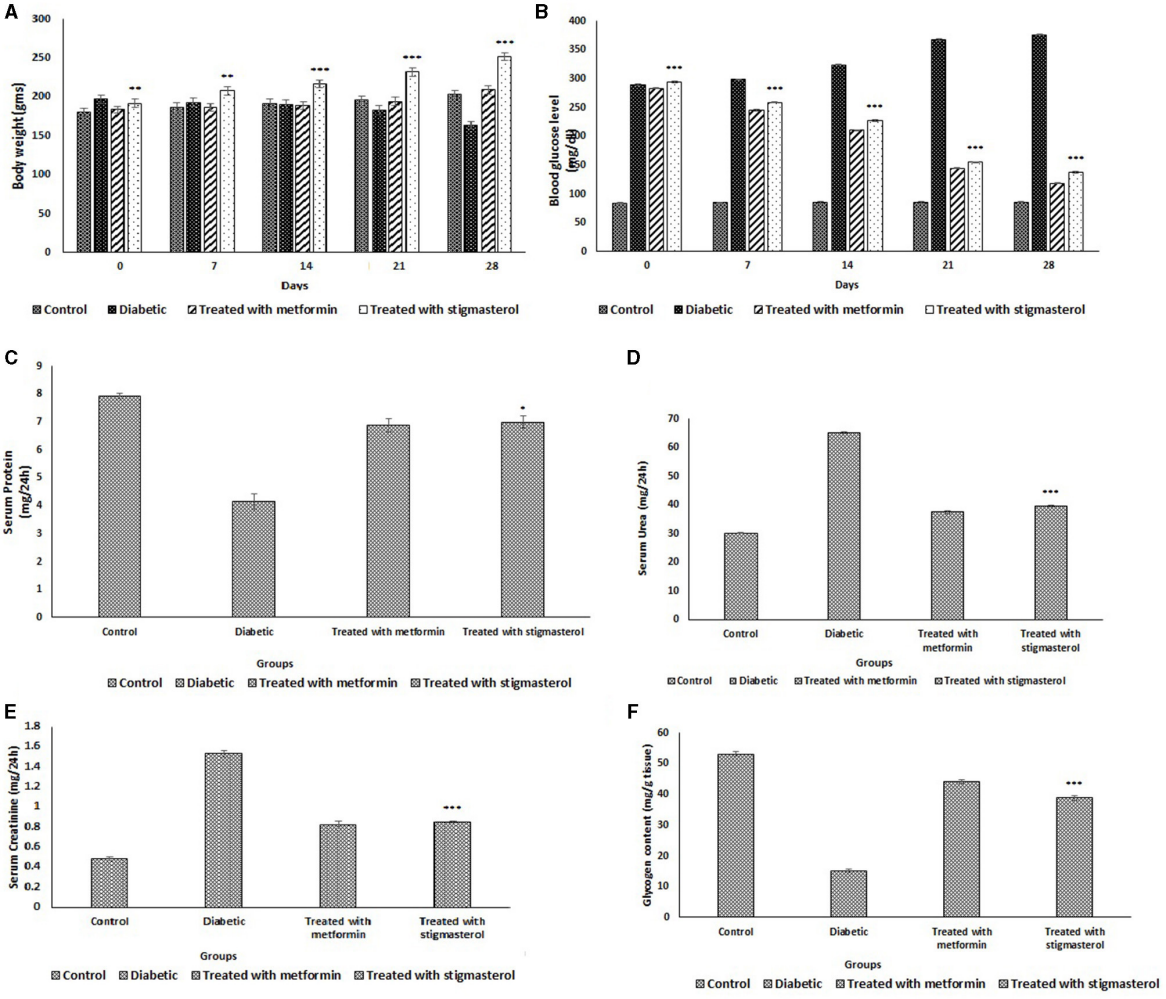
Effect of stigmasterol on rats **(A)** body weight (g), **(B)** blood glucose level (mg/dL), **(C)** serum protein (mg/24 h), **(D)** serum urea (mg/24 h), **(E)** serum creatinine (mg/24 h), and **(F)** glycogen content in control and diabetic rats. Number of animals used *n* = 10, error bars indicate standard deviations, significance *P < 0.05, **P < 0.01, and ***P < 0.001.

During the initial phase of experiment, the mean fasting blood sugar (FBG) level of groups 1- 4 were almost similar in the range of 84.12 ± 0.99 mg/dL. After 28 days, the FBG level was higher in the Group 2. The metformin- administered group showed reduction in the blood glucose level of 118.31 ± 1.1 mg/dL, while the diabetic group showed a glucose level of 376.12 ± 1.02. The stigmasterol administered rats (Group 4) showed a reduction in the FBG level of 137.73 ± 1.8 mg/dL when compared to the Group 2. The result obtained evidences when stigmasterol was administrated to the rats, lowering of blood glucose level was observed (Figure 4A).

The body weight of all the rat groups were in the range of 180 ± 5.2–197 ± 5.0 g at the starting day of treatment. The diabetic onset gradually reduced body weight in group-2, eventually to as low as 164 ± 4.8 g on an average after 28 days while the control rats weighed around 203 ± 4.9 g on an average. The Group 4 rats administrated with stigmasterol showed the average body weight of 252 ± 4.8 g, whilst the group 3 metformin administrated rats showed an average weight of 209 ± 5.9 g which was almost similar to that of control (Group 1) (Figure 4B).

The serum protein level was lower in diabetic rats (Group 2). Group 3 and Group 4 rats showed serum protein level almost nearer to control rats (7.92 ± 0.1 mg/24 h) (Figure 4C). Serum urea level was higher in the Group 2 rats with an average of 65.23 ± 0.2 mg/24 h which was much higher than the control (Group 1) rats of 30.21 ± 0.2 mg/24 h. The metformin (Group 3) and stigmasterol (Group 4) administrated rats showed the urea level of 37.56 ± 0.5 mg and 39.76 ± 0.3 mg/24 h (Figure 4D). Serum creatinine level for the control rats was on an average of 0.48 ± 0.02 mg/24 h while Group 2 showed an increased level of 1.53 ± 0.03 mg/24 h. The metformin and stigmasterol administrated groups showed almost similar values of 0.82 ± 0.04 mg and 0.85 ± 0.01 mg/24 h, respectively (Figure 4E). The average glycogen content was 53.1 ± 0.8 mg/g for control group. The untreated diabetic rats were 15.13 ± 0.7 mg/g which evidently depicts the glycogen storage was lower in diabetic rats due to their inefficiency in converting excess glucose to glycogen reserves due to impaired insulin secretion. The rats administrated with metformin and stigmasterol could reserve glucose as glycogen to a notable level of 44.25 ± 0.6 and 38.83 ± 0.8 mg/g, respectively. The administrations might have improved the insulin secretion level and thereby the glucose conversion to glycogen (Figure 4F).

### Histological Examination

The effects of stigmasterol on kidney, and liver of rats were evaluated. The control rats showed normal histoarchitecture and the histopathological examinations confirm the protective effect of stigmasterol (Supplementary Figure 1).

### Determination of Crystallinity and Particle Size Analysis of Nano-Emulsion

**Figure 5:**
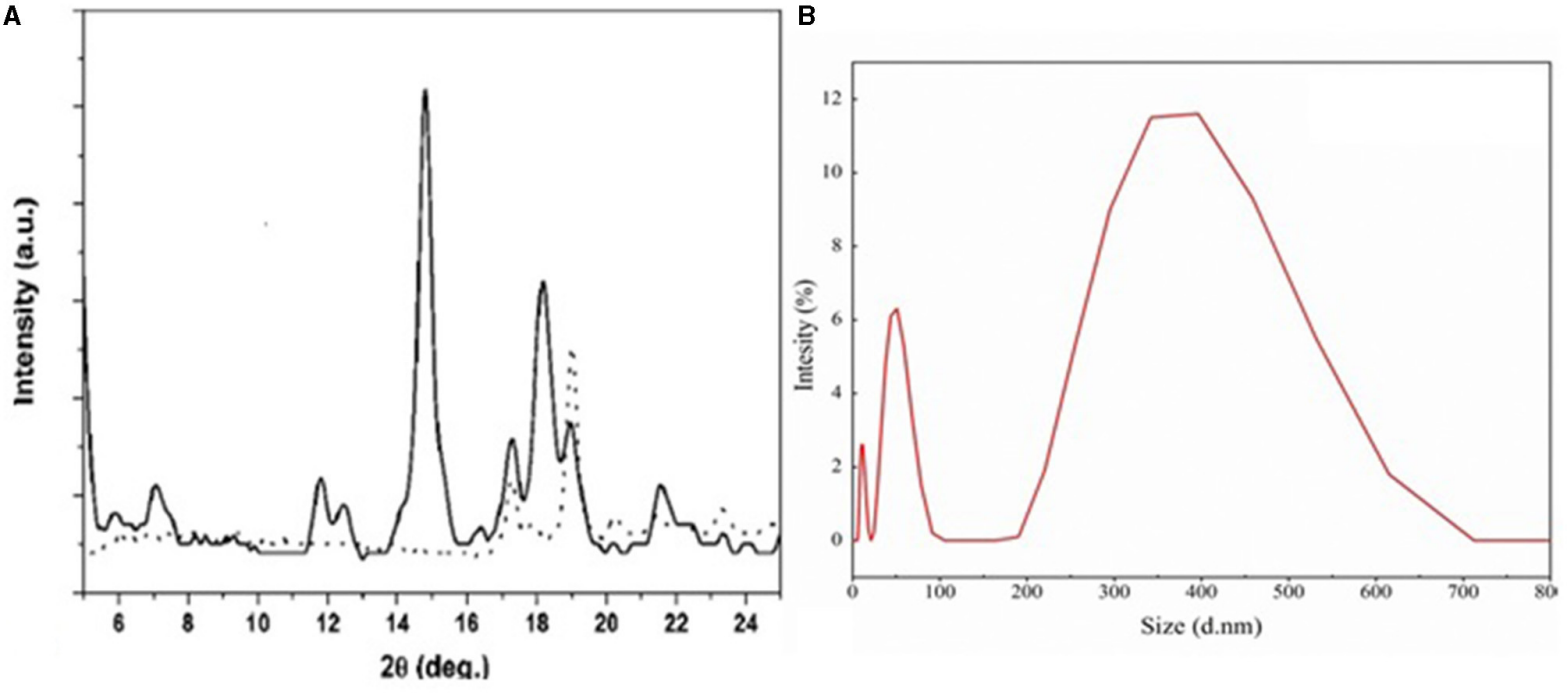
**(A)** Shows the nature of the particle determination by XRD, and the peaks obtained showed the particle were crystalline in nature. **(B)** Particle size analysis by particle size analyzer.

The crystallinity of the emulsion was confirmed by XRD analysis. Two high- intensity sharp peaks were obtained around 15°, 18.5° and small intensity peaks were observed at 11.9°, 12.19°, 16.2°, 19.2°, 21.9°, and 24° (Figure 5A). These peaks indicate the crystalline nature of the stigmasterol. Particle size analyzer showed the size distribution of particles of the stigmasterol was in the range of 50–500 nm (with a smaller intensity of 11.7 and 6.2%) (Figure 5B).

### Textural Characterization of the Stigmasterol- Incorporated Biscuit

Stigmasterol- incorporated biscuits showed a significant reduction in hardness when compared with the control. This may be due to the emulsified stigmasterol incorporated into the dough, which made them softer and due to the effect of stigmasterol on the starch protein interactions. The control biscuit showed hardness of 12.803 ± 1.8, with the reduced hardness in the stigmasterol incorporated biscuit 8.876 ± 2.3.

### Color Analysis of Biscuits

The color parameters were expressed as tri-stimulus attributes of L^*^, a^*^, and b^*^ values. The L^*^ value depicting lightness of 44.68 ± 0.11 in stigmasterol- incorporated biscuit when compared to control. The a^*^ value depicting red was almost similar for the control and stigmasterol- incorporated biscuit and the b^*^ value for yellowness was higher in the stigmasterol- incorporated biscuit when compared with that of control. The obtained result showed lightness and yellowness in stigmasterol incorporated biscuits compared with the control (Supplementary Table 1).

### Proximate Analysis of Biscuit

Stigmasterol incorporated biscuit showed reduced moisture and carbohydrate content. Higher values were observed in crude fiber, fat and protein content for the stigmasterol- incorporated biscuit (Supplementary Table 2).

### Sensory Evaluation of Biscuit

The sensory analysis showed incorporation of stigmasterol in biscuit production enhanced acceptance of the product. Color and appearance of the stigmasterol incorporated biscuit were evaluated in the range of “like very much” in comparison with “like moderately” of the control. The flavor, texture, crispiness and hardness were more acceptable for the stigmasterol incorporated biscuits when compared to the control. Overall acceptability of the stigmasterol biscuits was “like very much” whereas control was “like moderately” (Table 1).

**Table 1:** Comparison of sensory evaluation results of the stigmasterol incorporated and control biscuits.

**Sample**	**Color**	**Flavor**	**Texture**	**Appearance**	**Crispiness**	**Hardness**	**Overall acceptability**
Control	7.5 ± 0.68	7.2 ± 0.58	7.6 ± 0.63	6.9 ± 0.78	6.7 ± 0.84	6.9 ± 0.74	7.8 ± 0.75
Stigmasterol incorporated biscuits	8.0 ± 0.79	7.4 ± 0.72	7.7 ± 0.72	7.3 ± 0.65	7.2 ± 0.42	7.4 ± 0.58	8.2 ± 0.67

## Discussion

The seaweed *G. spinosum* was selected for the phytosterol extraction based on the wide distribution and abundance in the Mandapam coast, Rameswaram, India. Phytosterols such as β-sitosterol, campesterol, and stigmasterol are noted to play significant effects on lowering low density lipoprotein in plasma and they were found to be a safe nutraceutical (23, 24). The presence of stigmasterol in the extract of *G. spinosum* was confirmed by FTIR spectra and GC-MS showed stigmasterol with a retention time of 20.82 min and mass of 412.68 g/mol. In this study, phytosterol extract of *G. spinosum* showed a significant radical scavenging activity Methylene cholesterol, ergosterol and stigmasterol extracted from the algae *S. aggregatum* showed antioxidant activity (25). α- amylase inhibitory activity of the stigmasterol was evaluated, and the activity was found to be higher when compared with standard acarbose. The -glucosidase enzymes in the epithelial tissue of the small intestine are involved in the final phase of carbohydrate hydrolysis to produce an absorbable monosaccharide. Inhibition of these enzymes reduces postprandial hyperglycemia by slowing the breakdown of dietary polysaccharides into simpler saccharides in the gastrointestinal tract (26). *In vivo*, an experimental study on animals proved the anti-diabetic potential of stigmasterol with reduced blood glucose, urea and creatinine level. Administration of stigmasterol extracted from plants induces insulin uptake from pancreatic α-cells resulting antihyperglycemic effect (27–30). A banana extract containing 21.91% stigmasterol was found to have a possible anti-diabetic effect in alloxan-induced diabetic mice in a recent study (17). Similar to our study, metformin was used as a control drug. The mechanism of stigmasterol's anti-diabetic effect is not fully understood. However, certain putative mechanisms in antidiabetic potential have been identified. There are primarily either one of the two mechanisms involved, or a combination of both. The first mechanism, it could be due to a decrease in intestinal glucose uptake or an increase in glycolytic and glycogenic systems with a subsequent reduction in glycogenolysis and gluconeogenesis pathways, implying effects on the glucose metabolic pathways. The second mechanism involves the activation or repair of -cells, followed by insulin release or insulin receptor stimulation (17, 31, 32). In the present study, the stigmasterol administration reduced the fasting blood glucose level significantly in the diabetic rats, similar to commercial drug metformin. The anti-diabetic activity of stigmasterol may be due to the regeneration of the β-cells of Langerhans of the pancreas and thereby secretion of insulin, thus controlling the blood glucose level (29, 30, 33). Due to high plasma glucose, renal alterations occur resulting in high protein metabolism that leads to impaired nitrogen balance and eventually elevated levels of creatinine and urea levels which serve as a marker for kidney dysfunction (17, 34). Stigmasterol administration showed reduced creatinine and urea level when compared with the untreated diabetic rats, and the levels were almost similar to normal rats. There was a significant serum protein level reduction in diabetic rats when compared to other groups. This reduction might be due to the effect of insulin on maintaining the balance of protein by stimulating amino acid uptake and inhibition of degradation of protein (34, 35). Diabetic rats had low glycogen levels due to breakdown of glycogen reserves and absence of excess glucose conversion to glycogen due to impaired insulin production. Stigmasterol administration might have involved in the regeneration of pancreatic cells by releasing insulin and thereby controlling the glucose and glycogen levels to normal levels.

Stigmasterol has been reported to have potential with the possible mode of targeting the glucose transporter GLUT4 including increased translocation and expression of GLUT4 (36). The stigmasterol was emulsified and incorporated into the dough for biscuit preparation. Among the available types of functional foods, biscuits possess wider functions, and nutritional value. They absorb less moisture, ready to eat and easily available cereal and can be made into varying formulations, increased shelf life and they are economical. Biscuits are considered as the best nutraceutical for providing health benefits to the consumers in right proportions (37, 38). Stigmasterol added whole grain wheat biscuits lowered LDL cholesterol after 4 weeks of administration (39). Biscuit is a convenient food to deliver fortified food for daily intake and it is a food with wider acceptability for people of all ages. Stigmasterol added biscuits showed soft texture when compared to the control. This may be due to the emulsifier that weakens the gluten network resulting in reduction of dough cohesiveness and thus reduces the hardness. The sensory analysis evident that the stigmasterol- incorporated biscuit was much acceptable than the control.

## Conclusion

The present study describes the stigmasterol extraction from *G. spinosum* and the presence of stigmasterol in the extract of *G. spinosum* was confirmed by FT-IR and GC-MS analysis. The anti-diabetic effect of stigmasterol was confirmed using *in vitro* and *in vivo* studies. Results obtained from this study showed stigmasterol isolated from *Gelidium* sp. are rich in antioxidants and exhibit anti-diabetic activity. The biscuit formulated using stigmasterol showed high acceptability, and thus it can be used as a functional food supplement and can also be used as an alternative strategy to treat diabetes.

## Data Availability Statement

The original contributions presented in the study are included in the article/Supplementary Material, further inquiries can be directed to the corresponding author/s.

## Ethics Statement

The animal study was reviewed and approved by Institutional Animal Ethics Committee of Pondicherry University.

## Author Contributions

NP, AR, and AC performed the experiment, GP performed statistical analysis and AS contributed in writing the manuscript. GK designed the experiments. JS designed the manuscript. All the authors approved the manuscript.

## Conflict of Interest

The authors declare that the research was conducted in the absence of any commercial or financial relationships that could be construed as a potential conflict of interest.

## Publisher's Note

All claims expressed in this article are solely those of the authors and do not necessarily represent those of their affiliated organizations, or those of the publisher, the editors and the reviewers. Any product that may be evaluated in this article, or claim that may be made by its manufacturer, is not guaranteed or endorsed by the publisher.
