# Supplementary material for: Anti-diabetic Potential of a Stigmasterol From the Seaweed Gelidium spinosum and Its Application in the Formulation of Nanoemulsion Conjugate for the Development of Functional Biscuits
Source: Front Nutr. 2021 Sep 16;8:694362. doi: 10.3389/fnut.2021.694362 (PMC8481687; doi:10.3389/fnut.2021.694362)
Supplement: Supplementary file 1 [file Data_Sheet_1.docx]

**Anti-diabetic potential of a stigmasterol from the seaweed *Gelidium spinosum* and its application in the formulation of nanoemulsion conjugate for the development of functional biscuits**


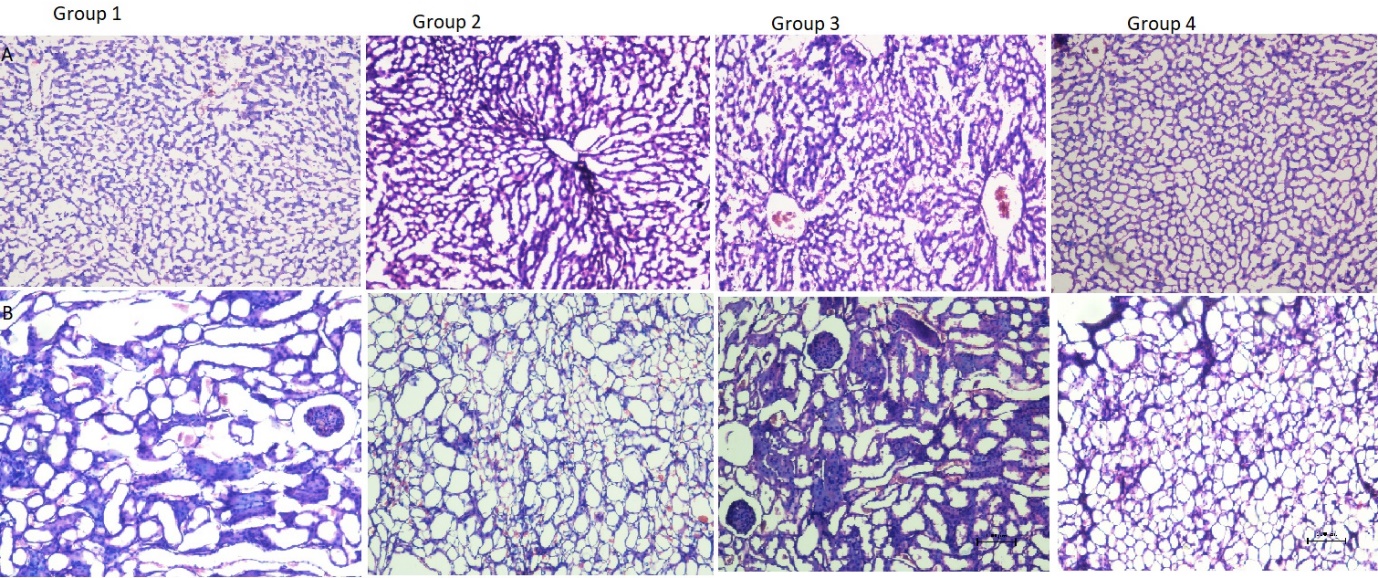


**Suppl. Fig.1** The results obtained showed protective effect of stigmasterol on the organ damage. The Group 1 rat showed normal kidney with glomeruli and tubules, blood vessels and interstitium appear normal. Group 2 indicates normal glomeruli, tubules showed focal mild epithelial thinning and vacuolation. Group 3 and Group 4 showed normal glomeruli and tubules. The section from liver showed normal morphology in group 1, with the evidence of normal hepatocytes and absence of inflammatory infiltrates. Group 2 shows dilated appearance with focal lymphocytic infiltrates. Group 3 showed distended architecture with mild inflammatory infiltrates. Group 4 showed intact architecture with normal hepatocytes. The stigmasterol showed better response similar to the control Group 1 rat.

Table 1: Comparison of the color analysis of the stigmasterol incorporated biscuits with control

| Sample | L* | a* | b* |
| --- | --- | --- | --- |
| Control | 39.44±0.07 | 15.19±0.04 | 26.97±0.08 |
| Stigmasterol incorporated biscuits | 44.68±0.11 | 15.13±0.03 | 30.75±0.09 |

Table 2: Proximate analysis of stigmasterol incorporated and control biscuits

| **Sample** | **Moisture** | **Ash** | **Crude fibre** | **Fat** | **Protein** | **Carbohydrates** |
| --- | --- | --- | --- | --- | --- | --- |
| **Control** | 2.48± 0.72 | 1.96± 0.87 | 0.54±1.12 | 29.28± 0.68 | 8.63± 0.93 | 57.11± 0.42 |
| Stigmasterol incorporated biscuits | 2.16± 0.54 | 1.98±0.68 | 0.59± 0.98 | 30.14± 0.48 | 8.71± 0.94 | 56.42± 0.38 |
